# Now you see it, now you don't: on emotion, context, and the algorithmic prediction of human imageability judgments

**DOI:** 10.3389/fpsyg.2013.00991

**Published:** 2013-12-26

**Authors:** Chris F. Westbury, Cyrus Shaoul, Geoff Hollis, Lisa Smithson, Benny B. Briesemeister, Markus J. Hofmann, Arthur M. Jacobs

**Affiliations:** ^1^Department of Psychology, University of AlbertaEdmonton, AB, Canada; ^2^Department of Linguistics, University of TuebingenTuebingen, Germany; ^3^Department of Psychology, Experimental and Neurocognitive Psychology, Dahlem Institute for Neuroimaging of Emotion, Free University BerlinBerlin, Germany

**Keywords:** emotions, context effects, imageability, lexical access, co-occurrence statistics

## Abstract

Many studies have shown that behavioral measures are affected by manipulating the imageability of words. Though imageability is usually measured by human judgment, little is known about what factors underlie those judgments. We demonstrate that imageability judgments can be largely or entirely accounted for by two computable measures that have previously been associated with imageability, the size and density of a word's context and the emotional associations of the word. We outline an algorithmic method for predicting imageability judgments using co-occurrence distances in a large corpus. Our computed judgments account for 58% of the variance in a set of nearly two thousand imageability judgments, for words that span the entire range of imageability. The two factors account for 43% of the variance in lexical decision reaction times (LDRTs) that is attributable to imageability in a large database of 3697 LDRTs spanning the range of imageability. We document variances in the distribution of our measures across the range of imageability that suggest that they will account for more variance at the extremes, from which most imageability-manipulating stimulus sets are drawn. The two predictors account for 100% of the variance that is attributable to imageability in newly-collected LDRTs using a previously-published stimulus set of 100 items. We argue that our model of imageability is neurobiologically plausible by showing it is consistent with brain imaging data. The evidence we present suggests that behavioral effects in the lexical decision task that are usually attributed to the abstract/concrete distinction between words can be wholly explained by objective characteristics of the word that are not directly related to the semantic distinction. We provide computed imageability estimates for over 29,000 words.

In the literature on word recognition and reading, *imageability* refers to the extent to which a word evokes a tangible sensation, a phenomenological effect that is traditionally measured by human judgment. Our goal in this paper is to outline the factors that contribute to imageability judgments. We review evidence suggesting that human imageability judgments are correlated with factors that have nothing to do with evoked sensation and demonstrate that the variability in imageability judgments and in some of their behavioral effects can be largely or entirely accounted for by measurable features that do not directly reference evoked sensation. The two features we focus on here are *the density of the contexts* in which the word occurs and *the emotional associations of the word*, with both measures derived from a co-occurrence model of semantics. As we review below, both of these measures have previously been associated with imageability. The unique contribution in this paper is how we measure them. Our goal is to show that when measured using objective formal methods (rather than subjective judgments), these measures account for essentially *all* of the variance in imageability judgments and their behavioral effects. Estimating imageability using algorithmic methods grounded in empirical measures has the practical benefit of allowing us to derive principled imageability estimates for every word in the English language.

## Background

Researchers distinguish *imageability* from *concreteness*, the extent to which a word's referent is amenable to detection by the senses and from other possibly related variables such as sensory experience ratings (Juhasz et al., 2011) or body interaction ratings (Paul et al., 2008). Although closely related, imageability and concreteness can dissociate. For example, the word *eternal* shows a large concreteness/imageability rating difference, with a medium imageability rating from (Wilson, 1988) of 4.03/7 (*z* = −0.27) and a low concreteness rating (2.14/7; *z* = −1.61), perhaps suggesting that judges have a tangible *feeling* about eternity. However, usually imageability and concreteness are highly correlated. Across the 1609 words for which we have both measures, they correlate at 0.64 [*t*_(1607)_ = 33.9, *p* < 0.00001].

Imageability differences have been shown to have many behavioral effects, including effects on recall (e.g., Paivio, 1971, 1985, 1995; Hamilton and Rajaram, 2001) and lexical access (e.g., James, 1975; Strain et al., 1995; Westbury and Moroschan, 2009). Children acquire imageable words earlier than non-imageable words (Bloom, 2000). Many aphasic patients perform better with imageable words than non-imageable words (e.g., Goodglass et al., 1969; Coltheart et al., 1980), though the opposite pattern has also been documented (e.g., Warrington, 1975; Warrington and Shallice, 1984; Sirigu et al., 1991; Breedin et al., 1995; Cipolotti and Warrington, 1995; Marshall et al., 1996; Papagno et al., 2009). Accessing imageable vs. non-imageable words evokes different brain activity (e.g., Friederici et al., 2000; Jessen et al., 2000; Fiebach and Friederici, 2003; Noppeney and Price, 2004; Binder et al., 2005).

The best-known theoretical account of imageability is Paivio's (1971, 1985) *Dual-coding theory*. Dual-coding theory suggests that imageable words have an advantage over non-imageable words because they are represented with two codes: a verbal code that can be used to represent words at both extremes of the imageability spectrum, and a non-verbal code that can be used to represent imageable words that also have sensory-motor information associated with them.

Another theoretical account, *context availability theory* (Schwanenflugel and Shoben, 1983; Schwanenflugel and Stowe, 1989; Schwanenflugel, 1991), proposes that imageability effects can be accounted for by a single system connecting words to their network of associated semantic knowledge. Behavioral differences in accessing words at either end of the continuum reflect differences in the amount of information that helps to link that semantic knowledge with each word. According to Schwanenflugel (1991) “contextual information may come either from the comprehender's knowledge base or from the external stimulus context” (p. 242). High imageable words have easy access to prior knowledge and do not benefit from associated contextual information for processing and comprehension. Low imageable words depend to a much greater extent on that contextual information. Context Availability Theory predicts that behavioral effects of imageability should be strongest when the words are presented in little or no context, and weaker when a more explicit context is provided. This prediction has been shown to be true (Schwanenflugel and Stowe, 1989).

These two accounts share an obvious and seemingly obligatory feature: they define imageability in terms of *semantics*. However, imageable words differ from non-imageable words in many other ways than their meaning. Low imageability words tend to occur less frequently than high imageability words. Across the 3697 words considered in this paper, the logged orthographic frequency of the word (from Shaoul and Westbury, 2010b) correlates with imageability at 0.19 (*p* < 0.00001). Reilly and Kean (2007) have documented many other non-semantic measures that differ between high and low imageability words, including stress patterns, number of letters, rate of consonant clustering, affixation probability, and neighborhood density (see also Westbury and Moroschan, 2009; Reilly et al., 2012).

The suggestion that imageability judgments reflect more than just semantics is supported by systematic patterns of apparent errors that can be seen in imageability judgments. Some words (like *eternal*) have mid-range imageability ratings despite the fact that they are not perceptible with our senses. Other examples of such words include *heaven* (imageability rating of 4.3/7, *z* = −0.04), *glory* (imageability rating of 4.17/7, *z* = −0.16), and *grace* (imageability rating of 4.1/7, *z* = −0.21). The classification of these words as being of mid-imageability despite their indisputable non-perceptibility forces us to confront the possibility that raters use other cues than just sensory perceptibility^1^.

Imageability judgment norms also include nouns that have very specific concrete referents but are rated low on imageability. For example, the word *astrolabe* is rated only 1.5/7 (*z* = −2.39) on imageability, although astrolabes have a distinct physical form that is dictated by their function, making them extremely imageable. Other examples of apparently too-low ratings of easily imageable nouns include *bough* (imageability rating: 2, *z* = −1.97), *stein* (imageability rating: 2.1, *z* = −1.89), and *aster* (imageability rating: 2.2, *z* = −1.80). Given that all of these words refer to concrete objects, we assume these ratings are low only because subjects did not know what the words' referents were. Imageability ratings appear to sometimes reflect concept familiarity or ease of concept accessibility rather than imageability *per se*.

In this we treat judgments as dependent measures, deconstructing the judgments into objective component features.

## Study 1: modeling imageability judgments

Imageability judgments for 3813 words were compiled from four sources: Bird et al. (2001); Cortese and Fugett (2004); Stadthagen-Gonzalez and Davis (2006), and Wilson (1988), itself a compilation from Paivio et al. (1968); Gilhooly and Logie (1980), and Toglia and Battig (1978). Where we had multiple judgments, we averaged them together, after normalizing them to a scale from 1 to 7. In total, 3697 of these words appeared in the dictionary of our co-occurrence model (described below), so we used those words. In order to be able to validate our conclusions on an independent data set, we randomly split this dataset into two halves containing about 1848 words each.

### Predictor set 1: word co-occurrence

Firth (1957) famously suggested, “You shall know a word by the company it keeps.” The famous dictum is particularly applicable to abstract words. Under the assumptions of both Dual Code Theory and Context Availability Theory, concrete words can have their meaning fixed in part by non-lexical semantics. A person can learn about the referents of material words by interacting with those things in the world. However, abstract words have no sensory context. As Context Availability Theory proposes, abstract words should therefore be more reliant on the linguistic contexts in which they appear.

One problem with Context Availability Theory is that “context” is a slippery word. There are many different ways to define what a word's context is, each of which may be appropriate for some purposes and none of which clearly presents itself as best for any particular purpose, let alone for all purposes.

In trying to formalize what we mean by “context,” we might simply define a word's context as the words that appear near to that word in some large corpus. Co-occurrence models (e.g., Lund and Burgess, 1996; Landauer and Dumais, 1997; Burgess, 1998; Shaoul and Westbury, 2006, 2010a, 2011; Jones and Mewhort, 2007; Rhode et al., 2007; Durda and Buchanan, 2008) eschew this first-order context and use *second-order co-occurrence* to define context. In co-occurrence models, words share a context not if they occur *together* (first order co-occurrence), but if they occur *with similar words* (second order co-occurrence). By looking at their co-occurrence contexts, we might be able to conclude that e.g., “semiotics” and “symbolism” had associated meanings even if the two words never occurred in proximity to each other.

In this paper we begin with a measure of context computed using the open-source model of word co-occurrence, HiDEx (Shaoul and Westbury, 2006). HiDEx computes a standard distance in co-occurrence space, the *neighborhood membership threshold*, that is a function of the mean and standard deviation of inter-word distances between billions of random word pairs (for details, see Shaoul and Westbury, 2006, 2010a). This threshold enables the definition of two measures of semantic density. *Average Radius of Co-Occurrence* (ARC) is the mean distance between the target word and all words within its threshold. *Neighbor Count* (NCOUNT) is the number of neighbor words within that threshold. We use a transformation of NCOUNT that we call *Inverse Neighbor Count* (INV-NCOUNT), defined as 1/(NCOUNT + 1), which correlates better with many behavioral measures (including imageability judgments) than NCOUNT itself does. We used the default parameter set shown in Table 1 (for justification, see Shaoul and Westbury, 2010a), computing co-occurrence measures from a corpus of approximately 12 billion words of USENET postings (Shaoul and Westbury, 2010c).

**Table 1:** **HiDEx parameter set for computing co-occurrence measures**.

**Corpus**	**UseNet Corpus**
Corpus size	12,714,502,395 words
Context size	10000 words
Window length behind	5 words
Window length ahead	5 words
Weighting scheme	Inverse Ramp
Normalization method	PPMI
Similarity metric	Cosine

For more details, see Shaoul and Westbury, 2010a,b,c, 2011.

Both ARC (*r* = 0.18, *p* < 0.00001) and INV-NCOUNT (*r* = −0.28, *p* < 0.00001) were reliably correlated with the imageability ratings for the 1848 words in the test set. A linear regression to predict the imageability ratings using only these two measures entered both predictors reliably (Table 2), and had an *r*^2^ value of 0.08 (*p* < 0.00001). This regression equation performed equally well on the separate validation set (*r*^2^ = 0.08, *p* < 0.00001).

**Table 2:** **Regression model for predicting human imageability judgments from two quantitative measures of context derived from co-occurrence similarity measures, on the test data set consisting of 1848 items**.

	**Estimate**	***SE***	***t*-value**	***p***
Intercept	2.26	0.81	2.80	0.005
ARC	2.59	0.82	3.13	0.002
INV-NCOUNT	−0.64	0.06	−9.93	<2e-16

*Multiple R^2^: 0.08; AIC: 5670 F*_(*2, 1845*)_ = *83.29, p* < *2.2e-16.*

We conclude from this initial analysis that, as suggested by Context Availability Theory, formally-defined measures of contextual density derived from co-occurrence models are highly reliable predictors of imageability judgments.

### Predictor set 2: emotional valence

Altarriba et al. (1999) and Kousta et al. (2011) (among others) have presented evidence suggesting that affective information (emotional association, which combines valence and arousal judgments; see Footnote 3 in Kousta et al., 2011) is more important for abstract than concrete concepts. Emotional association in these studies has been measured with human ratings. Here we developed an algorithmically well-defined measure of emotional association that relies only on co-occurrence measures.

We began by taking 78 distinct terms that have been proposed by different emotion theorists as “basic emotion terms.” These terms are summarized in Table 3 and, in slightly more detail, in Appendix 1. We then undertook a backwards regression using all 78 terms to predict the imageability judgments, removing at each step the term that predicted least well.

**Table 3:** **The sources and names of all “basic emotion” terms considered**.

**Source**	**Included terms**
Ekman et al., 1969	Anger, disgust, fear, happiness, sadness, surprise
Ekman, 1999	Amusement, anger, contempt, contentment, disgust, embarrassment, excitement, fear, guilt, happiness, interest, pleasure, pride, relief, sadness, satisfaction, shame, surprise
Kassam et al., 2013	Anger, disgust, envy, fear, happiness, lust, sadness, shame
Osgood et al., 1957	Active, bad, good, passive, strong, weak
Panksepp, 1982	Care, fear, lust, panic, play, rage, seeking
Plutchik, 1980	Anger, anticipation, disgust, fear, joy, sadness, surprise
Reizenzein, 2009	Aversion, desire, disappointment, fear, happiness, hope, relief, surprise, unhappiness
Robinson et al., 2004	Approach, arouse, away, danger, evaluate, from, safe, to, toward, withdraw
Stevenson et al., 2007	Anger, disgust, fear, happiness, sadness
Tomkins, 1962, 1963: Mild terms	Anger, contempt, distress, enjoyment, fear, interest, shame, surprise
Tomkins, 1962, 1963: Strong terms	Anguish, disgust, excitement, humiliation, joy, rage, startle, terror
Wundt, 1896	Depression, excitement, pleasant, relaxation, tension, unpleasant

For more details, see the Appendix.

Rather than stopping when all terms entered with *p* < 0.05, we continued until only eight terms remained, all of which entered into the regression with *p* < 1e-08. There were three reasons for this. One is that the average number of terms in the models we drew the terms from was eight. The second was that eight terms is a tractable number for human beings to easily consider. The third reason was that limiting the number of predictors to only the most highly predictive limits the likelihood of over-fitting, since we eliminate a lot of “detail” fitting with slightly contributing predictors. Stopping at *p* < 0.05 would have left us with 22 emotion term predictors that accounted for about 6% more variance in the test set, and about 3% less variance in the validation set than our 8-predictor set did.

The 8-predictor set is shown in Table 4. It included four terms with positive weights associated with higher imageability—*horny, pleasure, proud,* and *from*—and four with negative weights associated with lower imageability*—envious, admirable, arouse,* and *good*. These distances were very good predictors of the 1848 imageability judgments in the test set, with *r*^2^ = 0.31 (*p* < 0.00001). When the same regression equation was used to predict the 1849 imageability judgments in the validation set, it performed almost exactly as well (*r*^2^ = 0.30, *p* < 0.00001), suggesting that the equation was not over-fit to the test set.

**Table 4:** **Regression model for predicting human imageability judgments using co-occurrence distance from emotion terms, on the test data set consisting of 1848 items**.

	**Estimate**	***SE***	***t*-value**	***p***
Intercept	4.34	0.18	23.56	<2e-16
Admirable	−11.46	1.67	−6.87	8.96E-12
Arouse	−14.84	1.54	−9.61	<2e-16
Envious	−16.50	2.82	−5.85	5.87E-09
From	7.45	0.99	7.55	7.08E-14
Good	−11.31	0.93	−12.11	<2e-16
Horny	19.42	2.41	8.05	1.44E-15
Pleasure	13.17	1.10	11.93	<2e-16
Proud	11.16	1.56	7.17	1.12E-12

*Multiple R^2^: 0.31: AIC: 5161 F*_(*8, 1839*)_ = *102.4, p* < *2.2e-16*.

Although it is an excellent predictor of imageability judgments and validates well, the eight-emotion set has the disadvantage that it does not admit of any obvious theoretical interpretation. All but one of the emotion terms—*envious*—would normally be considered positively valenced. The set includes distances from the two closely-related (albeit not synonymous) terms *horny* and *arouse*, with opposite weights. Although a theoretically-grounded predictor set would be preferable, we note that our goal here is mainly practical, to be able to predict imageability judgments from well-defined objective measures.

Another concern about this model is that we began with such a large pool of predictors. It is possible that any group of 78 words might offer a rich enough set of possibilities to predict imageability judgments. In order to see if this was true, we made up 10 sets of 78 random words, drawn from all words of medium frequency (between 10 and 500 occurrences per million) in the Shaoul and Westbury (2010b) frequency norms. None of the 780 words was also contained in the 78 emotion predictors or the 3813 words for which we had imageability judgments. We repeated the same backwards regression as above, to predict imageability judgments on half the judged dataset beginning with each set of 78 terms and continuing to remove predictors until just 8 of those 78 remained. We then validated the regression equation on the other half of the judgment data.

The results are summarized in Table 5. Every one of the ten randomly-defined sets produced eight items that were strongly reliable predictors of the imageability judgments. This is perhaps not surprising, given the large number of predictors we began with. What is more surprising is that in every case the derived regression equation was also a highly reliable predictor of the *validation set* judgments, suggesting that the equations were not simply over-fit to the test set. This result implies that it is possible to reliably predict imageability judgments using selected words from *any set* of 78 word co-occurrence similarities.

**Table 5:** **Correlation of regression model estimates with imageability ratings, for ten models composed of eight words selected by backwards regression from 78 random words, and one model composed of eight words selected by backwards regression from 78 emotion terms, on both a development set of ratings and a separate validity set**.

**SET**	**TEST *r***	**VALIDATE *r***
1	0.57	0.60
2	0.58	0.57
3	0.63	0.71
4	0.58	0.57
5	0.57	0.59
6	0.59	0.61
7	0.58	0.54
8	0.58	0.60
9	0.58	0.57
10	0.57	0.58
EMOTION	0.55	0.55

**Figure 1:**
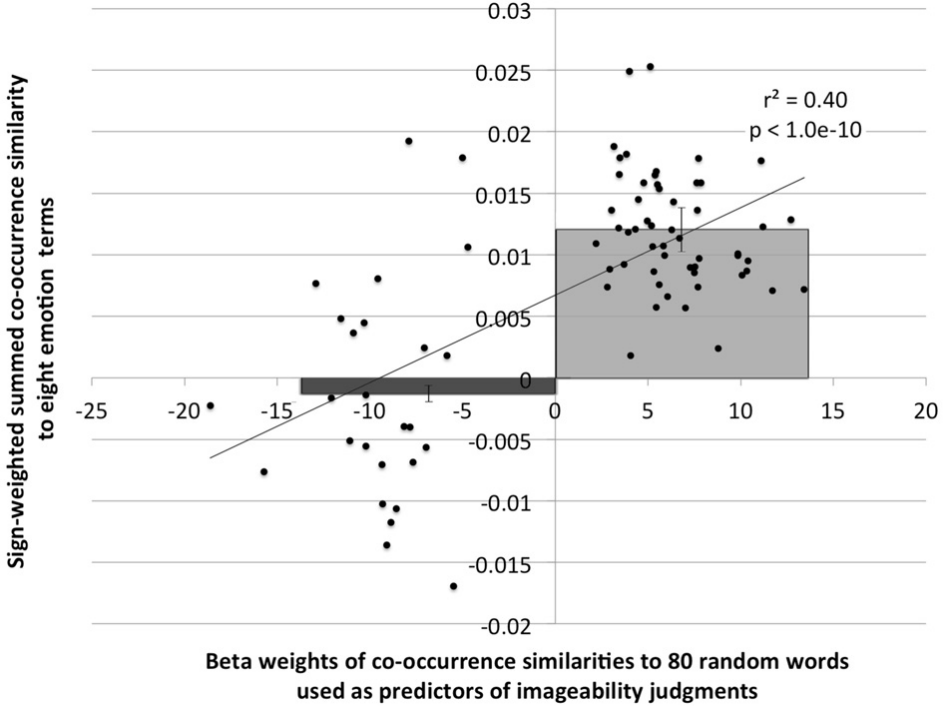
**Correlation between beta weights of 80 random word predictors from ten regression models of imageability, graphed against the sign-weighted summed co-occurrence similarity of each random word to eight emotion terms.** The overlain bar graph shows the average [SE] sign-weighted summed co-occurrence similarity for random word predictors that had negative or positive beta weights [*t*_(78)_ = 10.0, *p* < 9e-16].

This is a rather remarkable conclusion, which compels us to consider: what quality of a word both predicts imageability and is sufficiently universal that any random set of a few dozen words covers its range? Unsurprisingly, we think the answer is: *emotional valence*. To see if selected predictors were reflecting emotion valence, we looked at the correlation between the 80 beta weights from all ten random-word models, and the co-occurrence distance from the eight emotion terms in our best model, weighted positive or negative according to their weight in that regression model. As shown in Figure 1, these values are very highly correlated (*r* = 0.63, *p* < 1.0e-10) and the direction of sign on the beta weights of the 80 random words in the ten models divides the signed summed distance estimates into two distinct groups (*t*_(78)_ = 10.0, *p* < 9e-16; see overlay on Figure 1). This suggests that the predictor words selected from the ten sets of random words are serving as proxies for emotional valence.

Against this strict interpretation is that fact that some of the random sets reliably outperformed the original set of eight emotion terms in predicting imageability judgments, in both the test and validation set. It seems odd to say that a proxy for a measure should perform better as a predictor than the measure itself. The performance of the random sets suggests that our prediction set of eight emotion terms is sub-optimal for predicting imageability, at least for this particular dataset of imageability judgments. However, it has the virtue of being theoretically motivated and comprehensible as a generalization of the performance of the ten otherwise-unrelated random datasets, so we have continued to include it in our model.

### Predictor set 3: context, emotional valence, and lexical variables

When we added ARC, INVNCOUNT, LNFREQUENCY, word length (LENGTH), and orthographic neighborhood size (ON) to the eight-emotion model developed above, 10 of the 12 variables contributed reliably (*p* < 0.05), with ON and distance from the word *from* dropping out (Table 6). This full model had *r*^2^ = 0.36 (*p* < 0.00001; *AIC* = 5032) on the test set and again transferred very well to the validation dataset, with *r*^2^ = 0.36 (*p* < 0.00001). This appears to be a robust model of imageability judgments.

**Table 6:** **Full regression model for predicting human imageability judgments, on the test data set consisting of 1848 items**.

	**Estimate**	***SE***	***t*-value**	***p***
Intercept	3.25	0.72	4.51	7.00E-06
Admirable	−14.72	1.51	−9.75	<2.0e-16
Arouse	−12.83	1.42	−9.03	<2.0e-16
Envious	−9.93	2.78	−3.57	3.64E-04
Good	−9.00	0.82	−11.02	<2.0e-16
Horny	17.92	2.33	7.70	2.18E-14
Pleasure	11.49	1.08	10.65	<2.0e-16
Proud	7.47	1.55	4.84	1.44E-06
Arc	1.91	0.71	2.69	7.18E-03
Inv-ncount	−0.32	0.06	−5.27	1.55e-07
Length	0.07	0.03	2.54	0.011123
Lnfrequency	0.12	0.02	7.10	1.78E-12

*Multiple R^2^: 0.361: AIC: 5032 F*_(*11, 1836*)_ = *92.7, p < 2.2e-16.*

One of the advantages of developing models of imageability judgments based on non-subjective measures is that they can be easily extended to new words, since the objective measures we need to estimate the judgments are well-defined for all words. We have used the regression equation derived above to compute imageability judgments for 29018 English words, which we will make available^2^ at: http://www.psych.ualberta.ca/~westburylab/.

The 50 words estimated to be highest and lowest in concreteness are reproduced in Appendix 2. Although one might question a few of the high imageability words (are “adventures,” the second most imageable word, really more imageable than “fish,” the 34th most imageable word?), the lists generally cleave unambiguously to the expected distinction between the sensible and the non-sensible.

### Discussion

We have demonstrated that a large proportion of the variance in imageability judgments can be accounted for with three variable types: measures of co-occurrence density, measure of co-occurrence similarity to emotion terms, and some lexical measures that were previously known to be correlated with imageability judgments (word length and LNFREQUENCY).

We noted above that some words (citing *eternal, grace, heaven*, and *glory* as examples) seemed to be rated “too highly,” with average imageability ratings despite their obvious non-imageability. These words are also all estimated to be of mid or higher imageability using our regression equation, from −0.03z (for *grace*) to 2.0z (for *heaven*). The fact that we can replicate these “errors” suggests that judges may be also using affective cues rather than ontological knowledge to make their judgments.

Conversely, we had noted that several highly imageable words—citing *stein, bough*, and *aster*—seemed to be rated too low, close to −2z by subject ratings, and suggested that this was due to subject ignorance. Our method, which has information about how these words are actually used, correctly rates these imageable words as of at least average imageability (between −0.13z for *aster* and 0.46z for *bough*). We note that the average estimated imageability judgment across all words was well on the imageable side of the 7-point scale, at 4.3.

We have observed that there is a tendency for critics to assume that a model that predicts imageability judgments with a correlation of 0.60 (*r*^2^ = 0.36) accounts for just 36% of the variance that those judgments account for. Of course this is not the case since imageability judgments are themselves not perfectly correlated *with each other*. A model of a dataset cannot possibly exceed the inherent reliability of that dataset. From the sources listed above, we have at least two independent imageability judgments from independent sources for 1608 words. These judgments are correlated at 0.81. Setting *t*-values equal gives an equivalent correlation of 0.79 (*r*^2^ = 0.62) for 1849 items, the number of items in our test and validation sets. Our model therefore accounts for about 36.5/62, or about 59% of the variance that is accounted for by judgments from independent sources.

We are ultimately not interested in the judgments themselves so much as in how those judgments relate to behavioral measures. In the next section we consider how well our model predicts such behavioral measures.

## Study 2: predicting lexical decision reactions times

In this section, we demonstrate that the variance attributable to EMOTIONALITY and CONTEXT derived above predicts the same variance in lexical decision reactions times (LDRTs) as imageability does. We retrieved LDRTs for our 3697 words from the English Lexicon Project (Balota et al., 2007) and examined the ability of imageability to account for variance in the residuals before and after taking into account EMOTIONALITY and CONTEXT. If the predictors in our model account for the same variance as IMAGEABILITY, entering those predictors should eliminate the variability in the residuals that is attributable to IMAGEABILITY.

Entering ON, LENGTH, and LNFREQUENCY alone accounted for 29.1% of the variance in LDRTs in the full set of 3697 words. Using IMAGEABILITY to explain the remaining variance (i.e., to explain variance in the residuals) accounted for 6.1% of the variance in those residuals before removing the variance attributable to CONTEXT and EMOTIONALITY (*p* < 0.00001). After removing the variance in the residuals that is attributable to CONTEXT and EMOTIONALITY (which interacted reliably, *p* = 0.009), IMAGEABILITY accounted for 57% as much variance (3.5%), still a highly reliable amount (*p* < 0.00001).

### Study 2 discussion

Overall, the results above support the claim that the predictors in our model are accounting for a large proportion of the variance that is usually attributed to IMAGEABILITY.

## Study 3: analysis of word distributions

There is an additional facet to our claim that imageability judgments can be explained by EMOTIONALITY and CONTEXT, which is that the distributions of these two predictors are not uniform across the range of imageability. In this section we provide the evidence to support this claim, and explain why it is important for understanding any behavioral differences attributed to IMAGEABILITY.

**Figure 2:**
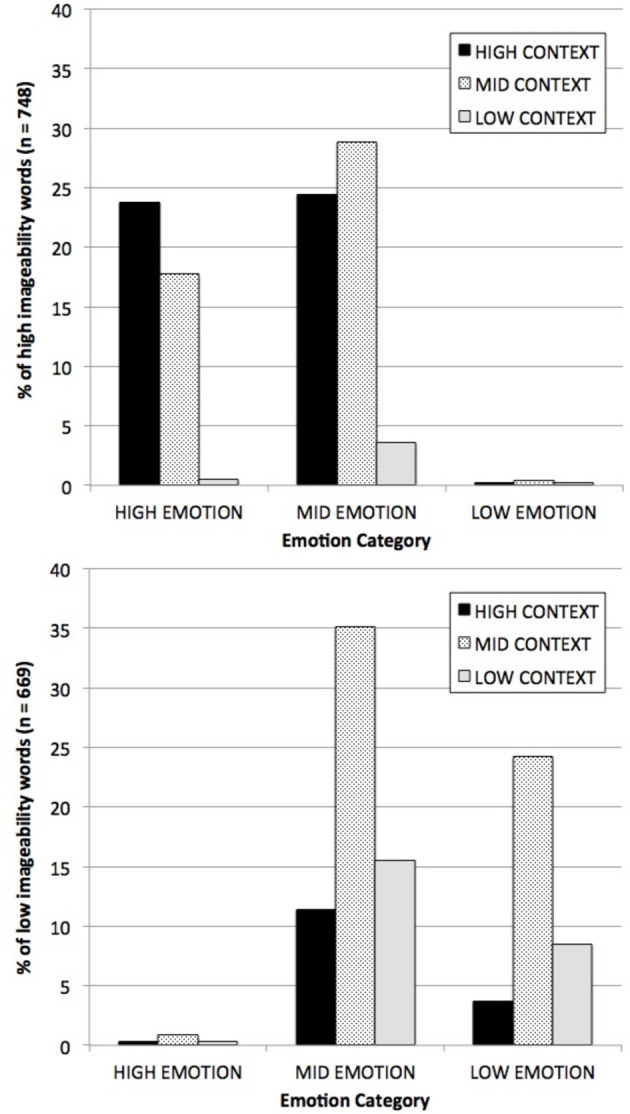
**Distribution of high imageability words (top) and low imageability words (bottom), by CONTEXT and EMOTIONALITY category.** “High” and “Low” categories along each dimension were defined using a ±1 SD cut-off.

To illustrate the problem, we normalized the values of EMOTIONALITY and CONTEXT (as defined by the regression estimates of each dimension, considered above) and split the measures into three groups along each dimension: high (>1z), low (<1z) and medium (everything in between). The percent of high and low imageability words that fall into each of the nine categories defied by crossing these two categories is shown in Figure 2, which clearly shows how unevenly the words are distributed. There are, for example, 80 times more high emotion/high context words among the high imageability words (23.8% of all high imageable words) than among low imageability words (0.30% of all low imageable words). Conversely, there are 32 times as many low emotion/low context words among low imageability words (8.5% of all low imageable words) than among high imageability words (0.27% of all words). Fully 95% of all high imageability words fall into the mid or high range on both dimensions, vs. just 48% of all low imageability words.

Of course this means that when we divide our words up into high and low imageability categories based on human judgments, we are going to be sampling in unequal proportions from the nine categories of high/mid/low CONTEXT × high/mid/low EMOTIONALITY. If the average RT for each category is different, the magnitude of these disproportions will have a strong effect on imageability category RTs.

**Figure 3:**
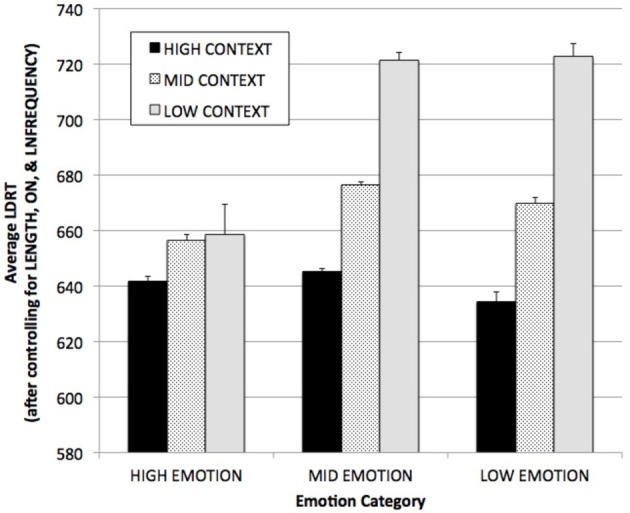
**Average English Lexicon Project (Balota et al., 2007) LDRTs for words in each of the nine CONTEXT × EMOTIONALITY categories, after controlling for ON, LNFREQUENCY, and LENGTH.** “High” and “Low” categories were defined using a ±1 SD cut-off. Bars are SE.

The average LDRTs for words in each of the nine CONTEXT × EMOTIONALITY categories (after co-varying out the variance attributable to LENGTH, ON, and LNFREQUENCY) are shown in Figure 3. Words in the high/high category (i.e., the words that are 80 times more common among high imageability than low imageability words) have average (SE) estimated RTs of 642 (1.7) ms, while words in the low/low category (that are 32 times more common among low imageable than high imageable words) have average (SE) estimated RTs about 80 ms slower, 723 (4.7) ms. More generally (covering the entire spectrum of words) words that are high on either context or emotionality (which make up 67% of all high imageability words, but just 17% of all low imageability words) are responded to over 50 ms faster on average (647 ms) than words that are not high on either dimension (698 ms), which make up 83% of low imageability words but just 33% of high imageability words.

This has an obvious result: when we select our words by high/low human imageability judgment, we are making very skewed selections across CONTEXT and EMOTIONALITY. If we assume that there is no imageability effect at all (i.e., use the global average RT in each CONTEXT × EMOTIONALITY subtype, collapsed across all words), and simply draw words proportionally to their observed frequency from among the high and low imageability groups (after co-varying out the variance attributable to LENGTH, ON, and LNFREQUENCY), we would find that our “high imageability group” had an average RT of 658 ms, while our low imageability group had an average RT of 680 ms: that is, we would find an “imageability effect” of 22 ms that is founded on the assumption that there is actually no difference in RTs attributable to imageability *per se*.

This is approximately equal to the actual imageability effect in this dataset. After again co-varying out the variance attributable to LENGTH, ON, and LNFREQUENCY, the average (SE) RT for all high imageability words is 657 (1.4) ms, vs. 682 (2.0) ms for low imageability words, a difference of 25 ms. Almost all (22/25 or 88%) of the imageability effect is actually explicable by the two predictors we are considering here.

We suspect that in most psychological experiments the effects of CONTEXT and EMOTIONALITY will be much more extreme than they are in this global analysis that includes medium imageability words, since experimentalists often tend to draw from the extreme ends of their measures, where the disparities of both proportion and RT are also at their extremes. For example, the estimated RT difference between high emotion/high context (“high imageability”) words and low emotion/low context words (“low imageability”), again after controlling for the three lexical variables, is 81 ms.

To see if this was so in the real world, we collected data in a lexical decision experiment using a previously published stimulus set that we knew to be tightly controlled.

## Study 3: lexical decision

### Participants

Our participants were 65 undergraduates (23 males; 42 females), with an average (SD) age of 18.8 (1.6) years and an average (SD) of 13.6 (1.0) years of education. All reported themselves to be right-handers with normal (or corrected-to-normal) vision who had learned to speak English before the age of 6. They participated in return for partial course credit as part of a subject pool at the University of Alberta.

### Stimuli

We used the stimulus set published in Binder et al. (2005), which had been constructed (by Westbury) without any regard for emotionality. That stimuli set was controlled using phonological data and frequency counts from the CELEX lexical database (Baayen et al., 1995). It consisted of 100 low to medium frequency nouns (with an orthographic frequency between 2 and 170 per million words) and 100 non-words, all from four to six letters in length. Half of the words had low imageability ratings (217–436 on a 700 point scale) and half had high imageability ratings (range: 577–644). The imageability ratings of these words were very strongly correlated with concreteness ratings at *r* = 0.94 (*p* < 0.0001). The concrete and abstract words were matched to each other and to the non-words on letter length, phoneme length, mean summed and minimal positional bigram frequency, and orthographic neighborhood. The matching was done algorithmically on an item-by-item basis so all matched pairs were close on all of these dimensions simultaneously. Additionally, the words were also matched item-by-item to each other on CELEX orthographic frequency.

In the original 2005 paper that used these stimuli, we reported a 33 ms imageability effect, with average RTs of 740 ms for the high imageability word, and average RTs of 773 ms for the low imageability words (*F*_(1, 23)_ = 15.72, *p* < 0.0001). Because of the small number of participants and the fact that the original data were collected in an fMRI scanner, we collected new data.

### Procedure

The experiment was a simple VLD experiment. Subjects were told that they would see letter strings on the screen, and that their task was to decide as quickly as possible whether the string was a word (“c” key) or a non-word (NW) (“x” key). They were asked to use the first and second fingers of their right hand to press the response keys. The first two stimuli shown were practice stimuli that were included only to accustom them to the paradigm and that were never analyzed.

Data were collected using ACTUATE software (Westbury, 2007b) on Apple G4 Macintosh Minis connected to 17” LCD monitors. Strings were presented on the screen in 60-point Times font, in the middle of a white rectangle measuring 102 × 312 pixels, which was centered on the screen against a black background.

### Results

The average (SD) correct decision rate in the standard lexical decision condition was 92.7% (4.7%). Two subjects were eliminated for having percent correct scores more than 2.5 SDs below the average (76% and 79%), leaving an average of 93.2% correct scores. All erroneous and NW responses were then removed and discarded. Sixty-six responses (2.1%) were removed because they were too quick, under 400 ms. Nine responses (0.3%) were removed for being too slow, over 4000 ms. After excluding these responses, all remaining responses that were more than 2.5z outside of the average of the remaining stimuli were removed, eliminating an additional 89 responses (2.8%) for being too slow.

The RT data were analyzed using generalized linear mixed-effect regression models, fitted by Laplace approximation (see Baayen, 2008). LENGTH, ON, LNFREQUENCY, CONTEXT, EMOTIONALITY, and IMAGEABILITY were treated as fixed effects. Subjects, stimulus order, and stimulus were treated as random effects. Models were compared using Akaike Information Criteria (AIC) values, which offers a principled criterion that allows comparison of models on “goodness of fit” (minimization of information loss in the model) while taking into account the number of parameters. *P*-values were calculated using a Monte Carlo Markov chain (MCMC) random walk with 10000 simulations.

## Results

The average (SE) RT for correct responses to high imageability words was 629 (12.3) ms, vs. an average (SE) RT of 684 (14.6) ms for low imageability words, for an apparent imageability effect (before controlling for any of the lexical variables, which were, however, tightly matched between imageability categories in this dataset) of 55 ms [*F*_(1, 124)_ = 8.3, *p* = 0.004].

We used linear mixed effects models to assess the effects of the predictors. We began with a base model that included ON, LENGTH, LNFREQUENCY and random effects of subject, string, and stimulus order. Neither ON nor LENGTH contributed reliably (*p* > 0.05, presumably because the stimuli set consisted of uniformly short words), so they were dropped from the base model. The resultant base model had an AIC value of 38712.

When EMOTIONALITY and CONTEXT were added to this model, only EMOTIONALITY (which correlated with IMAGEABILITY at *r* = 0.84 across all words in the experiment, *p* < 0.00001) contributed reliably. The model with this predictor added had an AIC value of 38700, suggesting that it was markedly better than the base model (hundreds of times more likely to minimize information loss).

With the contributions of these predictors fixed, we entered IMAGEABILITY. It did not contribute reliably (*t* = −0.32, *p* = 0.75). As predicted, the two factors (or, in this case, just one factor) we have considered in this paper accounted for all the variance that had been attributed to IMAGEABILITY.

## Discussion

This result is entirely consistent with the analyses above, which showed that IMAGEABILITY is largely predictable from CONTEXT and EMOTIONALITY, that CONTEXT and EMOTIONALITY categories are distributed disproportionally among the high and low imageability words, and that the expected RTs in the CONTEXT × EMOTIONALITY categories vary widely and systematically across the range of those two predictors.

This result is also consistent with imaging evidence. In the functional imaging study of lexical decision of abstract vs. concrete words that used the same stimulus set, Binder et al. (2005) identified activity in left posterior prefrontal cortex (BA11) as being strongly associated with low-imageability words. The authors noted that this region had been linked with phonological processes, verbal short-term memory, and lexical retrieval (Démonet et al., 1992; Paulesu et al., 1993; Warburton et al., 1996; Fiez et al., 1999) and interpreted the activation in those terms. However, BA 11 has also been strongly linked to representing the affective valence of a stimulus (Knutson et al., 2001; O'Doherty et al., 2001), especially in ways that are sensitive to changes in motivational context (Bechara et al., 2000; Rolls, 2000; Ochsner and Feldman Barrett, 2001; for a review and discussion, see Ozier and Westbury, 2013). The low-imageability word activity documented in the Binder et al. study may therefore be also interpreted as being consistent with a key proposition of this paper that words in this class are being assessed in large part by having stronger emotional associations than high imageability words.

These neuroanatomical considerations lead us to speculate that the rating task used to collect imageability norms may not be executed in the way that the users of the task have always assumed. When a participant is instructed to rate words according to the extent which each “arouses a mental image, i.e., a mental picture or sounds, or other sensory experience” (the instructions used in Cortese and Fugett, 2004), it is normally assumed that they attempt to mentally sense the word and then report how easy or successful that attempt was. The ubiquitous, unconscious influence of our emotional system on cognitive processes leaves open the possibility that the subject's mind is also (perhaps implicitly) considering a different question: “Do I really *want* to image that word?” The rating that the participant provides might include an emotional component so that introspective feeling of judging sensibility reported is lower for words that are not desirable to sense (e.g., *blood*) and higher for words that are desirable to sense (e.g., *heaven*).

This is consistent with work focused specifically on the role of emotional valence in lexical access, which has found (except at the highest levels of negative valence) a negative correlation between emotional valence and RT: e.g., faster RTs for more pleasant words (Kuchinke et al., 2005, 2007; Kanske and Kotz, 2007; Schacht and Sommer, 2009; Silva et al., 2012). However, we note that imageability is correlated with many other lexical measures as well, including concreteness, (discussed above) and action-relatedness (Paul et al., 2008) and the presence of visual features (Hauk et al., 2008). Precisely because they are so highly inter-correlated, it is very difficult to adjudicate on the relative contribution of these individual variables to RT or human judgments and the present study cannot do so definitively.

## Conclusion

We have taken two routes to trying to explain imageability. In the first section of this paper, we reported success in predicting the imageability with a single measure each of contextual density and emotionality. In the second section, we verified that our derived model of imageability accounted for most or all of the variability in LDRTs attributed to human ratings, both in a large database and in a replication of a previous experiment. We also showed how systematic differences in the distribution of the components of our model underlie RT differences.

The claim that LDRT effects attributed to imageability manipulations can be explained by CONTEXT and EMOTIONALITY does not of course nullify or cast any doubt upon the results of previous experiments that have used IMAGEABILITY manipulations: it rather contributes to *explaining* the effects obtained in those experiments. Effects attributable to human judgments are not altered or nullified because we offer an explanation in terms of objective measures of what may underlie those human judgments. However, the evidence does suggest that we need to be cautious in attributing causal efficacy in lexical access to the construct of imageability, *per se*. Effects attributed to the imageability category, as determined by human judgments, can be largely or wholly accounted for by using objective predictors that do not include any measure of whether a word's referent has features that are accessible to the senses.

In closely related work (Westbury, 2013) we have recently shown that a very simple model of emotion (based on co-occurrence distances from just the six terms Wundt's (1896) model) can also account for *all* the variance that is usually attributed to human judgments of subjective frequency (i.e., estimates derived from a co-occurrence model correlate with subjective frequency judgments as well as independently-collected subjective frequency judgments). This kind of unification of disparate phenomena under the same explanatory framework is an important piece of supporting evidence. The fact that two different kinds of human lexical judgments (which are—as Westbury, 2013; Westbury, explicitly discusses—hardly correlated) can both be explained using emotionality estimates gives us some confidence that these measures may have explanatory efficacy.

In presenting this work, we have been asked if it is our intention to replace human imageability judgments with our own measures. We have shown that our measures correlate strongly with human imageability judgments; that they are as good as human imageability judgments in the limited domain of predicting LDRTs; that they have qualitative “face validity”; and that they can be algorithmically extended to a large set of words. These are all arguments in their favor. However, we of course do not pretend to have solved the problem of grounding imageability judgments definitively. The empirically-grounded predictors of human imageability judgments we consider here are surely proxies for affective information that might be better defined with more neurobiologically relevant, more predictive, more widely applicable, or more theoretically-enmeshed predictors that will thereby be an improvement on ours.

Nothing in this paper should be taken to imply that there are no semantic differences between imageable and non-imageable words. However, we offer compelling reasons to doubt that the behavioral differences attributed to imageability in the lexical decision task have anything to do with those semantic differences.

### Conflict of interest statement

The authors declare that the research was conducted in the absence of any commercial or financial relationships that could be construed as a potential conflict of interest.

## Footnotes

^1^It may also be that the religious nature of these particular examples has exposed people either to imaginative pictorial representations of the concepts, or caused them to ponder them deeply enough to feel they can almost sense them (a point we will return to). If so, the examples still pose a problem for our traditional understanding of what it means to be of “low imageability.”

^2^Note that we have to exclude the eight emotion terms that actually appear in the model, because their self-similarity of 1 gives them highly skewed estimates.

## Appendix 1

### Sources of the basic emotion terms

Wundt's (1896) proposed that emotion consisted of three basic axes: one for evaluation (“pleasant/unpleasant”), one for arousal (“excitement/depression”) and one for attention (“tension/relaxation”).

Osgood et al. (1957) used factor analysis on a large set of affective ratings to derive three binary dimensions: “good/bad,” “active/passive,” and “strong/weak.”

Tomkins (1962); Tomkins (1963) proposed eight basic emotions (ignoring his non-lexicalized ninth emotion, dismell, or reaction to a bad smell), defining the dimensions with two terms each, the first for the milder version of the emotion and the second for a stronger related emotion: “interest/excitement,” “enjoyment/joy,” “surprise/startle,” “distress/anguish,” “fear/terror,” “shame/humiliation,” “contempt/disgust,” and “anger/rage.”

Ekman et al. (1969) proposed seven basic emotions: happiness, surprise, fear, sadness, anger, and disgust. Ekman (1999) extended this list with an additional eleven terms: amusement, contempt, contentment, embarrassment, excitement, guilt, pride, relief, satisfaction, pleasure, and shame.

Plutchik (1980) derived eight primary emotions from consideration of evolutionarily adaptive emotions relevant across species: anger, fear, sadness, disgust, surprise, anticipation, and joy.

Building on the four basic emotions (expectancy, rage, fear, and panic) he had originally argued for in 1982, Panksepp (2005) proposed that neural hard-wiring in the mammalian brain underlay seven primary emotions: seeking, fear, rage, lust, care, panic, and play.

Robinson et al. (2004) analysis of emotion focused on the general emotional characteristics of arousal and valence. They did not suggest a specific set of labels for basic emotions, so we took the general focus of their analysis and created a set of labels related to danger or the lack thereof, to arousal, and to approach/avoidance behavior: approach, avoid, toward, away, to, from, evaluate, arouse, danger, and safe.

Stevenson et al. (2007) extended the affective norms (valence and arousal judgments) collected by Bradley and Lang (1999), for five emotions dimensions that they considered to be cross-culturally universal (following Ekman, 1993; Levenson, 2003): happiness, sadness, fear, disgust, and anger.

Reizenzein (2009) proposed that most or all emotions could be expressed as variants of just a few: happiness, unhappiness, hope, fear, surprise, disappointment, and relief. Analysis of these emotions depends in Reizenstein's theoretical framework on desire and aversion, so we also included these two terms.

Kassam et al. (2013) looked at the neural correlates of nine basic emotions: anger, disgust, envy, fear, happiness, lust, sadness, and shame. They also listed 18 related terms, of which we added 17: angry, enraged, disgusted, envious, jealous, afraid, frightened, happy, joyous, lustful, horny, proud, admirable, sad, gloomy, ashamed, and embarrassed. Their term revulsed did not appear in the HiDEx dictionary and was not included in our list.

## Appendix 2

The 50 words estimated to be highest and lowest in imageability, from a set of 29,018 words. Imageability ratings have been normalized.

**Table:**

**HIGH IMAGEABILITY**	**zESTIMATE**	**HIGH IMAGEABILITY**	**zESTIMATE**
fishing	3.56	engender	−4.63
adventures	3.40	lot	−4.26
trees	3.40	ethic	−4.17
river	3.39	inflame	−4.15
mediterranean	3.34	better	−4.15
boats	3.34	incite	−3.99
wooden	3.33	inspire	−3.99
mountain	3.31	abominate	−3.99
boat	3.30	imply	−3.95
pink	3.25	sincere	−3.87
beach	3.21	provoke	−3.84
champagne	3.20	tenacity	−3.74
decorated	3.19	rile	−3.74
kitchen	3.17	honesty	−3.71
springs	3.16	poise	−3.68
restaurant	3.15	honest	−3.63
girl	3.08	courage	−3.62
chocolate	3.08	lack	−3.60
lake	3.07	lacking	−3.58
miniature	3.06	arouses	−3.58
dog	3.06	toughness	−3.57
tree	3.04	certainly	−3.53
water	3.04	sense	−3.52
restaurants	3.03	patience	−3.52
yellow	3.02	tamp	−3.50
spotted	3.01	intentioned	−3.46
dancing	3.01	instil	−3.44
rock	2.99	innate	−3.37
purple	2.98	twinge	−3.31
adventure	2.98	kind	−3.29
gardens	2.97	alienate	−3.29
sea	2.97	reflects	−3.28
park	2.96	impressed	−3.27
fish	2.95	teamwork	−3.26
ocean	2.94	allay	−3.26
trailer	2.94	galvanize	−3.26
flowers	2.93	sincerity	−3.26
daughter	2.92	elicit	−3.24
monkey	2.91	wetly	−3.24
band	2.90	solid	−3.23
courtyard	2.90	lacks	−3.22
turtle	2.90	understandable	−3.22
candy	2.89	arousing	−3.20
nearby	2.88	decisiveness	−3.19
room	2.88	instincts	−3.16
plastic	2.88	ineducable	−3.15
queen	2.87	smirked	−3.15
boulevard	2.87	pragmatism	−3.15
golden	2.87	fostering	−3.14
floating	2.87	aroused	−3.14
